# Disabled women's attendance at community women's groups in rural Nepal

**DOI:** 10.1093/heapro/dav099

**Published:** 2015-10-30

**Authors:** J. Morrison, T. Colbourn, B. Budhathoki, A. Sen, D. Adhikari, J. Bamjan, S. Pathak, A. Basnet, J. F. Trani, A. Costello, D. Manandhar, N. Groce

**Affiliations:** 1 Institute ofGlobal Health, University College London, 30 Guilford Street, London WC1N 1EH, UK; 2 Mother Infant Research Activities (MIRA) Nepal, PO Box 921, Thapathali, Kathmandu, Nepal; 3 Leonard Cheshire Centre for Disability and Inclusive Development, Department of Epidemiology and Public Health, University College London, 1-19 Torrington Place, London WC1E 6BT, UK

**Keywords:** participation, access, community health promotion, population health, public health intervention

## Abstract

There is strong evidence that participatory approaches to health and participatory women's groups hold great potential to improve the health of women and children in resource poor settings. It is important to consider if interventions are reaching the most marginalized, and therefore we examined disabled women's participation in women's groups and other community groups in rural Nepal. People with disabilities constitute 15% of the world's population and face high levels of poverty, stigma, social marginalization and unequal access to health resources, and therefore their access to women's groups is particularly important. We used a mixed methods approach to describe attendance in groups among disabled and non-disabled women, considering different types and severities of disability. We found no significant differences in the percentage of women that had ever attended at least one of our women's groups, between non-disabled and disabled women. This was true for women with all severities and types of disability, except physically disabled women who were slightly less likely to have attended. Barriers such as poverty, lack of family support, lack of self-confidence and attendance in many groups prevented women from attending groups. Our findings are particularly significant because disabled people's participation in broader community groups, not focused on disability, has been little studied. We conclude that women's groups are an important way to reach disabled women in resource poor communities. We recommend that disabled persons organizations help to increase awareness of disability issues among organizations running community groups to further increase their effectiveness in reaching disabled women.

## INTRODUCTION

There is broad consensus that communities should be actively involved in improving their own health. As the 1978 Alma Ata Declaration states: ‘people have the right and duty to participate individually and collectively in the planning and implementation of their health care’ (WHO, 1978). Many believe that participatory approaches to health can empower communities to take responsibility for diagnosing health and development problems, and work together to solve them (Morgan, 2001).

In this article, we critically examine our participatory approach in rural Nepal, to understand the participation of disabled women in our intervention. Our research group at the Institute for Global Health, University College London (UCL) has been working with Mother Infant Research Activities (MIRA) Nepal to evaluate the impact of participatory women's groups on maternal and newborn survival. A recent meta-analysis of data from cluster randomized controlled trials of similar interventions found that women's group intervention areas experienced significant reductions in maternal and newborn death. When more than a third of pregnant women participated in groups, maternal deaths fell by 55% and newborn deaths fell by 33% (Prost *et al.*, 2013). Often, interventions fail to reach the most marginalized (Gwatkin, 2003), and therefore we have been working with the Leonard Cheshire Centre for Disability and Inclusive Development at UCL to understand disabled women's participation in women's groups, and other community groups. It is estimated that people with disabilities constitute 15% of the world's population and face disproportionately high rates of poverty, stigma, social marginalization and unequal access to health resources (WHO and The World Bank, 2011, Hosseinpoor *et al.*, 2013, Mitra *et al.*, 2013). Women's groups may provide an important source of empowerment, social support and information for disabled women in low-income countries.

## WOMEN'S GROUPS IN NEPAL

The MIRA/IGH collaboration conducted a cluster randomized controlled trial of women's groups, from 1 November 2001 until 31 October 2003 in 24 clusters (Village Development Committees) of Makwanpur District. There was a decrease in neonatal mortality by 30% in 12 intervention clusters when compared with 12 control clusters (Manandhar *et al.*, 2004). The intervention and surveillance systems have been described elsewhere (Osrin *et al.* 2003a,b, Morrison *et al.*, 2005, 2010a,b; Morrison, 2011). Women's groups were convened once a month by a local female facilitator, who was not a health worker. She led the group through a participatory learning and action cycle of problem identification, community planning to address identified problems and working together to implement and evaluate plans. After this initial trial (Phase I), we ran women's groups in all 24 clusters, from 1 November 2003 until 31 December 2008 (Phase II). Groups did not specifically target inclusion of disabled women, but were marketed and perceived to be open to all married women.

## QUANTITATIVE METHODS

Makwanpur District is in the central development region, south of Kathmandu. It has a population of ∼420 500 (Central Bureau of Statistics, 2012), and there are over 16 different ethnic groups residing there. Almost half the population are of disadvantaged Tibeto-Burman Buddhist Tamang ethnicity, and the second largest ethnicity being the more advantaged Hindu Brahmin/Chhetris. Makwanpur is a hilly district with 83% of the population people engaged in subsistence farming, and it has a Human Development Index score of 0.497, which is slightly above the national average of 0.458 (UNDP and Government of Nepal, 2014).

To identify disabled women, we completed a screening questionnaire with married women enrolled in Phases I and II, who had had a baby while living in women's group intervention clusters. Due to migration since trial surveillance, we located 13 687 (77.6%) out of 17 628 women, assessed their disability status, and the severity and type of disability.

### Screening questionnaire

Disability was defined as being disabled through interaction between an individual's impairment and surrounding attitudinal and environmental barriers (UN, 2006). We adapted a questionnaire used in the National Disability Survey in Afghanistan (Trani and Bakhshi, 2008). It has 34 items on a four-point Likert scale ranging from 1 to 4 with increasing activity limitation or functioning problem and detects physical, sensory, learning, behavioural, neurological and psychological disabilities based on the International Classification of Functioning, Disability and Health (ICF) (WHO, 2001) and Sen's capability approach (Sen, 1999). The questionnaire captured severity of disability by asking respondents to rank their abilities on a four-point scale. We obtained verbal consent. When women found it difficult to answer questions, family or neighbours assisted.

The questionnaire was translated from English to Nepali, and back translation confirmed quality. Fourteen experienced researchers received two days training on the tool and completed 100 pilot interviews. We revised the questionnaire at a review meeting. These researchers trained and supervised 43 locally employed interviewers. Data were collected from October 2010 to April 2011 by 26 male interviewers, and 17 female interviewers. Researchers observed 740 (5.3%) interviews.

We assessed the internal consistency of the four-point scale across all 34 items in the screening tool and found an acceptable level of reliability based on the standardized Cronbach's alpha (0.777). Standardized Cronbach's alpha was used because we assumed that all scale items had equal variance (Terwee *et al.*, 2007; Tavakol and Dennick, 2011). The standard error of measurement (SEM) was 0.96, where a low SEM indicates high reliability. The minimal detectable change was 2.66 on a scale of 0–136. We assessed the psychometric properties of the screening tool for reliability and validity. Fifty-three women were interviewed twice in the same day by different interviewers to test for inter-rater reliability, and again after 10–14 days for test–retest reliability. We used Kappa measures of agreement for item-per-item inter-rater reliability and found: Kappa of 1 (identical responses) for 8 questions, Kappa ranging from 0.7 to 1 (good agreement) for 17 questions, between 0.5 and 0.7 (acceptable agreement) for 5 questions; question 23 had a value below 0.5. Question 23 asks about an aspect of mental illness, and responses were different for two women. Further research may improve understanding of the question and responses. We calculated inter-rater reliability using a weighted kappa of the overall scale and found a kappa coefficient of 0.79 showing a good strength of agreement (Terwee *et al.*, 2007). This result was confirmed by the calculation of a Bland–Altman plot: only 6% of the points were outside of the limits of agreement (Bland and Altman, 1983). We computed test–retest reliability of the overall scale using Pearson bivariate correlations and showed that there was a statistically significant correlation (*R* = 0.53, *p* < 0.001) from Day 1 to Day 14. We computed item per item test–retest reliability and found a correlation coefficient of 1 for 17 items, over 0.6 for 9 items and 0.5 for remaining items. We also calculated the weighted kappa (ICC) for test–retest and this was 0.688.

Identifying who is disabled is a complex process (WHO and The World Bank, 2011). The Central Bureau of Statistics in Nepal found that 8467 (2%) out of a population of 420 477 in Makwanpur had a disability—matching the national average—of which 3792 (44%) were women (Central Bureau of Statistics, 2012). Physical disabilities (2996) were the most common, with blindness/low vision (1553) and deafness (1340) being the second and third most prevalent disability type. However, these estimates may be conservative. Using the screening questionnaire, we found 29% (3930) of women with children had a mild, moderate, severe or very severe disability (Institute for Global Health, MIRA and Leonard Cheshire Disability and Inclusive Development Centre, 2013).

Participation is also difficult to measure (Rifkin *et al.*, 1988). As a crude measure of participation, we used a question from our women's group surveillance questionnaire that asked if a woman had ever attended a MIRA women's group (Phases I and II), and if yes, how many times (Phase II only). We calculated the percentage of women in clusters with women's groups who ever attended a group in each phase for all women and by severity and type of disability. Disability tends to increase with age, therefore we tested for significant observed differences with and without age as a confounding variable. We tested for significance of observed differences between non-disabled women and each severity level of disability, and type of disability using logistic regression. We also calculated number of meetings attended for Phase II women by severity and type of disability. We tested the significance of the observed differences from non-disabled women using Poisson regression, again both with and without the woman's age included as a confounder. Finally, using the larger Phase II sample, we examined how all women, disabled women (all categories of severity combined) and non-disabled women who attended groups and who did not, differed in terms of age, ethnicity, education, household assets and asset wealth quintiles. We have merged sight and hearing impairments into one disability type (sensory disability), as few participants had these disability types. We classified ethnicity into four categories: Tamang, Brahmin/Chhetri; other disadvantaged groups and other advantaged groups. All quantitative analyses were done in Stata 12.1 for Mac.

## QUALITATIVE METHODS

We purposively sampled four study clusters where we had been running women's groups and where there were more moderately and severely disabled women. We selected two hill clusters, and two plains clusters, to consider physical access issues.

We completed semi-structured interviews with 20 moderately and severely disabled MIRA group attenders and 20 non-attenders (Table 1). We also conducted two focus groups—one with Supervisors of the MIRA women's groups, and one with female community health volunteers (FCHVs), who had attended and helped to co-ordinate groups. Two female Nepali researchers collected data. They had been trained in conducting research with disabled women. Participants were approached in their homes and gave verbal consent. When necessary, a friend, neighbour or family member helped interpret or translate.

**Table 1:** Characteristics of women in the qualitative sample

Characteristics	Number of women
MIRA women's group attender	20
MIRA women's group non-attender	20
Topography of participants homes	
Hill	20
Plains	20
Type of disability	
Learning and developmental	13
Multiple disabilities	9
Physical	6
Sensory	5
Behavioural Psychological	4
Intellectual	3
Severity of disability	
Moderate	16
Severe	24
Ethnicity	
Brahmin/Chhetri	8
Tamang	24
Newar	3
Dalit and other marginalized castes	5
Total	40

Researchers located 19 severely disabled women, and 21 disabled women were identified as moderately or severely disabled through our screening tool. Data were recorded and transcribed directly into English. One recording was lost. Thematic content analysis was completed, coding data according to emergent themes (Green and Thorogood, 2005).

Ethical approval was obtained from the Nepal Health Research Council.

## QUANTITATIVE RESULTS

In Phase I clusters, 29.7% of 2916 women attended a women's group meeting (Table 2). Despite some variation, the proportion of disabled women attending groups was not significantly different to the proportion of non-disabled women (data not shown). This was true for each severity and type of disability except those with physical disabilities who were less likely to attend (odds ratio: 0.57, 95% CI: 0.35, 0.93). In Phase II clusters, 19.5% of 5770 women attended a women's group meeting (Table 2). In Phase II, there were also no significant differences in the proportion of disabled women in each level of severity and only the proportion of women with sensory disabilities (odds ratio: 1.45, 95% CI: 1.05, 2.00, *p* = 0.023) and those with neurological disabilities (odds ratio: 2.03, 95% CI: 1.15, 3.57) were more likely to attend meetings. Women with all other types of disability were as likely as non-disabled women to have attended at least one meeting. In Phase II, women attended an average of 1.15 meetings (SD: 4.37). Non-disabled women attended an average of 1.09 meetings (4.37). Significantly different attendance incidence was only found in mildly disabled women who attended 1.50 meetings (SD 5.27, *p* < 0.000, Table 2). When we examined type of disability, significant differences were found for women with learning disabilities, women with neurological disabilities and women with multiple severe or very severe disabilities who attended slightly more meetings than non-disabled women on average (1.37 meetings, *p* < 0.000; 2.64, *p* < 0.000, and 1.54 meetings on average, *p* < 0.000, respectively) (Table 2). Given that only 56 women reported neurological disabilities and 39 women reported multiple severe or very severe disabilities, the latter two differences could be spurious. The significance of the regression models remained the same when mother's age was included in the equation; except for multiple moderate where the coefficient indicating lower attendance becomes more significant, from *p* = 0.050 to *p* = 0.016.

**Table 2:** Women's group attendance for all women and by severity and type of disability

Phase I: 1 November 2001 to 31 October 2003 (attendance in 24 women's group clusters)	All women (*n* = 2916)	Not disabled (*n* = 1863)	Severity				Physical (*n* = 106)	Sensory (*n* = 286)	Learning (*n* = 205)	Behavioural (*n* = 107)	Epilepsy (*n* = 38)	Multiple mild (*n* = 158)	Multiple moderate (*n* = 35)	Multiple severe or Very severe (*n* = 17)
			Mild (*n* = 543)	Moderate (*n* = 341)	Severe (*n* = 128)	Very severe (*n* = 41)								
Ever attended a woman's group	29.7%	30.2%	30.0%	26.7%	30.5%	26.8%	19.8%	31.5%	30.7%	32.7%	34.2%	26.0%	22.9%	17.7%
Phase II: 1 November 2003 to 31 December 2008 (attendance in 24 women's group clusters)	*n* = 5770	*n* = 4131	*n* = 742	*n* = 542	*n* = 269	*n* = 86	*n* = 170	*n* = 209	*n* = 448	*n* = 340	*n* = 56	*n* = 162	*n* = 67	*n* = 39
Ever attended a woman's group	19.5%	18.9%	21.6%	20.3%	21.9%	18.6%	22.4%	25.4%	19.2%	17.9%	32.1%	21.6%	22.4%	20.5%
Number of meetings attended														
0	80.5%	81.1%	78.4%	79.7%	78.1%	81.4%	77.7%	74.6%	80.8%	82.1%	67.9%	78.4%	77.6%	79.5%
1	4.3%	4.2%	4.9%	4.2%	5.2%	2.3%	4.7%	5.3%	4.2%	3.5%	3.6%	6.2%	7.5%	0%
2	3.2%	3.4%	3.0%	2.0%	3.4%	3.5%	4.1%	5.3%	2.7%	1.8%	3.6%	1.9%	4.5%	0%
3	3.3%	3.2%	3.2%	4.1%	4.5%	1.2%	4.7%	3.8%	3.6%	2.7%	8.9%	1.9%	6.0%	0%
4 or 5	3.0%	3.2%	2.6%	2.8%	2.2%	3.5%	2.4%	3.4%	2.2%	1.2%	5.4%	4.3%	0%	7.7%
6–10	3.1%	2.6%	4.7%	4.2%	3.0%	5.8%	4.1%	4.3%	3.1%	5.9%	3.6%	4.9%	0%	10.3%
11 or more (max = 70)	2.5%	2.3%	3.2%	3.0%	3.7%	2.3%	2.4%	3.4%	3.4%	2.9%	7.1%	2.5%	4.5%	3.6%
Mean	1.15	1.09	1.50	1.16	1.21	1.10	1.26	1.30	1.37	1.17	2.64	1.20	0.90	1.54
SD	4.37	4.37	5.27	3.39	3.75	3.05	5.12	3.44	4.99	3.43	6.86	3.51	2.64	3.41
Poisson regression IRR (95% CI) compared with non-disabled^a^			1.33 (1.25, 1.42)	0.99 (0.91, 1.08)	1.06 (0.95, 1.19)	1.13 (0.92, 1.38)	1.02 (0.89, 1.18)	1.10 (0.98, 1.25)	1.38 (1.27, 1.50)	0.96 (0.86, 1.06)	2.62 (2.23, 3.09)	0.97 (0.84, 1.12)	0.78 (0.60, 1.00)	1.69 (1.31, 2.18)
*p*-value			<0.000	0.867	0.283	0.247	0.720	0.116	<0.000	0.387	<0.000	0.696	0.050	<0.000
Significance			****	ns	ns	ns	ns	ns	****	ns	****	ns	ns	****

IRR, incidence rate ratio; CI, confidence interval; ns, not significant.

^a^Significance does not change when mother's age is added to the regression equation; except for multiple moderate where the coefficient indicating lower attendance becomes more significant—from *p* = 0.050 to *p* = 0.016.

*p < 0.05, **p < 0.01, ***p < 0.001, ****p < 0.0001.

### Socio-demographic and socioeconomic differences

The average age of group attenders, and non-attenders was the same (28.5 years old and 28.4 years old, respectively). Disabled women had a higher average age than non-disabled women (30.3), but this did not differ between attenders and non-attenders (Table 3). When examining ethnicity, disability and group attendance, we found that fewer Tamang women and more women of Brahmin/Chhetri ethnicity had ever attended a group meeting among both non-disabled and disabled women. More Brahmin/Chhetri women, and more women of disadvantaged ethnicities were disabled overall, and fewer Tamang women were disabled.

**Table 3:** Socio-demographic characteristics of non-disabled and disabled women who have, or have never attended a women's group

	Not disabled		Disabled	
	Did not attend group (*n* = 3351)	Attended group (*n* = 780)	Did not attend group (*n* = 1294)	Attended group (*n* = 345)
Age				
Mean	28.4	28.5	30.3	30.3
SD	5.7	5.8	6.9	6.9
Ethnicity				
Tamang	67.4%	60.6%	60.2%	56.2%
Brahmin and Chhetri	15.6%	19.0%	20.3%	22.0%
Other advantaged group	7.8%	10.4%	8.4%	8.1%
Other disadvantaged group	9.2%	10.0%	11.2%	13.6%
Education				
No education	56.2%	50.1%	58.8%	53.9%
Up to class 5	24.1%	25.9%	23.4%	22.9%
Up to class 9	13.8%	15.1%	12.1%	15.4%
Class 10 pass or above	5.9%	8.9%	5.6%	7.8%
Literacy				
Unable to read	46.8%	39.1%	48.4%	39.7%
Reads with difficulty	17.2%	19.4%	17.9%	20.9%
Reads with ease	36.0%	41.5%	33.8%	39.4%
Assets				
Electricity	51.1%	53.3%	61.5%	56.5%
Radio	68.4%	73.7%	66.9%	64.9%
Television	20.3%	23.9%	26.7%	24.6%
Bicycle	8.8%	9.0%	10.2%	9.0%
Telephone	4.1%	8.1%	4.4%	6.7%
Asset quintiles				
Poorest	22.8%	17.7%	19.8%	19.7%
Pecond	56.2%	57.7%	53.7%	58.0%
Middle	13.0%	15.3%	18.0%	13.3%
Next-rich	5.9%	5.9%	6.6%	6.1%
Richest	2.1%	3.5%	1.9%	2.9%

Women who had ever attended a women's group were more educated and more literate than those who had not. These differences were similar among disabled and non-disabled women. Although disabled women were on average, less educated and less likely to be literate than non-disabled women (Table 3).

Five household assets (electricity, radio, television, bicycle and telephone) were used to construct an asset index and split the women into wealth quintiles. Non-disabled women in the richest four quintiles were more likely to attend groups; but among disabled women the relationship between asset index and group attendance was less clear (Table 3).

## QUALITATIVE RESULTS

We present data of how disability affects women and their access to community groups, and the perceived benefits of groups. Some women, usually those who were identified by the screening tool as moderately disabled with learning and developmental disabilities, reported being unaffected by their disability. However, most women felt that disability affected their daily life. Quotes are presented by a woman's type of impairment and whether they attended a MIRA women's group or not.

### Discrimination and mistreatment

Many disabled women reported discrimination and mistreatment from families and community members: ‘After marrying when I came to husband's house, my mother-in-law tortured me. She didn't give me enough food. She also beat me’ (non-attender, multiple disabilities). Another commented: ‘There are many people who, by their words show pity on (disabled persons), but inside their heart they think that we got what we deserved … . There are many people who hate us’ (non-attender, sensory disability).

FCHVs and some disabled women felt that their disability became worse as a result of this mistreatment: ‘The forgetfulness happens because of mental stress … . It is because of domestic violence. Some disabled women behave like that, and they can't even think about anything else because they are being tortured by their family’ (FCHVs).

Some disabled women felt it was their fate to be disabled. FCHVs told us that women disabled since birth were treated worse than women disabled through accident or illness. Being born disabled was often considered to be the result of ‘the past (bad) actions that her forefathers did’ (FCHVs). Supervisors and some women also reported that women with mental health or learning disabilities were treated worse than women with physical and sensory disabilities: ‘If people cannot walk then community members behave differently. If there is any kind of mental problem, then they tease them instead of showing concern’ (attender, learning and development disability).

Women with mental impairments were often believed to be possessed by a spirit or were thought to be foolish or stupid: ‘Mentally disabled women are blamed for their character by the community. If someone is forgetful then she is thought to be stupid, foolish or dim … people are not counted as mentally disabled until they are totally insane …  . those with general disabilities are not counted as mentally sick’ (Supervisors).

A woman believed to be mentally ill due to spirit possession can be feared or revered: ‘Some people are said to possess the divine spirit within themselves … some people are claiming to be a goddess and being worshipped as a goddess. Are they mentally disabled?’ (Supervisors).

Supervisors felt that disabled women were treated worse than disabled men: ‘Still now in our society, there is a tradition of underestimating women, and this is also true in the case of disabled women. People discriminate between men and women.’ Many women in our sample reported being married very young. Supervisors reported a belief that ‘hysteria’ ‘will be cured if (a woman) gets married.’

### Guilt and worry

Often women felt guilty that their disability limited or prevented them from working to support their family: ‘When the work is not done, or not done how it should be, it creates more tension … It's very difficult. I feel bad’ (attender, learning and developmental disability). Family and community members often ridiculed them because of this: ‘I can't bend, I can't work. I wish I could do all the housework but I have to make others work … people keep saying that I am taking advantage of my family members because of my impairment’ (attender, physical disability).

Many disabled women were worried about their future: ‘It is difficult. I can't walk. I do a little work. My daughters are doing work for me now but I don't know what will happen in the future’ (attender, physical disability).

### Access to groups

Many disabled women attended groups. Out of our sample of 40, 7 only attended MIRA groups, 13 attended MIRA and other groups, 9 only attended a savings group and 11 women did not attend any groups. Many who were not attending any community groups at the time of the study had been to a savings group in the past. Usually, group attendance was affected by several factors, and many of these were also common to non-disabled women.

#### Physical access and distance

Surprisingly, disabled women did not feel that physical access, or distance were major barriers, even among physically disabled women. Most women attended community groups close to their homes. ‘It's easy for me to reach the (savings group) meeting. It's near here’ (non-attender, multiple disabilities). In contrast, FCHVs and Supervisors felt that physical access and distance were barriers for disabled women.

#### Self-confidence

Another barrier to group attendance was lack of confidence. Many women said it was difficult for them to understand the discussion, and to speak in front of others: ‘I didn't know how to talk. What to say and how to say?’ (attender, physical disability). Women sometimes blamed their lack of education when explaining non-attendance in groups, or why they found it difficult to interact at first: ‘If I was able to do calculations then it would be easy for me, but I can't even write my own name. What's the purpose of going to the group if I can't easily understand everything? That's why I didn't go to the group’ (non-attender, learning and developmental disability). FCHVs stated that some ‘women felt uneasy coming to the group’, because they have not been to school and had little experience with group interaction. Supervisors believed that women's lack of understanding about the benefits of groups and the importance of attending groups was another reason for not attending: ‘Women are pessimistic about the group regarding what a group gives them, what they will understand, what significance it bears and why they should come to the groups.’ Yet this issue did not emerge from interviews with disabled women, indicating that perhaps families of disabled women lack understanding of benefits, as opposed to women themselves.

#### Family support

In our study, most disabled women going to groups were either not living in extended families or had supportive families that encouraged them to attend. Family support was necessary for a woman to be able to attend a group, whether she was disabled or not. A family needed to believe that they could benefit if the woman attended a group, and she usually needed to seek permission, ‘I talked to my family, and when they said yes I went to the agriculture group’ (attender, multiple disabilities). Those with unsupportive families said that their family did not believe there was value in encouraging them to attend. Many disabled women were perceived as incapable of learning or understanding. Women with all types of disability often felt under-estimated by families and community members: ‘My disability has affected me. People underestimate me’ (non-attender, learning and developmental disability).

Supervisors suggested that families might restrict the movement of disabled women because they were perceived to be more vulnerable: ‘Women who can't speak or can't see may be the victim of rape at any time. The families of women who are mentally disabled have to be alert all the time. They always have to watch over her. They have to take special care of her … .’

#### Poverty

Poverty may disproportionately affect disabled women. Poor disabled women doing farm work or daily wage labour had no time to come to the group. Women in nuclear families also found it difficult to leave the house: ‘There was no one at home, I was all alone. I could only go to the group after finishing all my housework’ (attender, learning and developmental disability).

Supervisors and women told us that participation in savings groups depends on community perceptions about their ability to pay back loans. If a woman has money, she can participate regardless of her disability. As one visually impaired woman said: ‘At present money is everything … If you have money then you can do anything, and if you don't have money then you can't do anything’ (non-attender, sensory disability). Several non-attenders reported that poverty prevented their attendance at savings groups: ‘(group members) are wealthy but I am not. I cannot put money in the savings group on time. So, why should I go to the group?’ (non-attender, psychological behavioural disability).

Other disabled women reported leaving savings groups having been unable to pay back their loan on time or leaving groups when they were unable to continue their contributions: ‘Friends invited me and they forced me to stay once I was in the group … When I had to deposit money in the group I didn't have any money. It was easy for me before but it's not easy for me now’ (non-attender, multiple disabilities). This made some community and group members angry and caused women considerable stress.

#### Barriers to MIRA group attendance

Some disabled women did not know about the MIRA group, or felt they already attended too many groups. Older disabled women felt the group was not relevant for them as they were unlikely to become pregnant. Other disabled women, particularly those with learning or hearing disabilities said they were unable to follow discussions and preferred to attend skills development or savings groups: ‘What to say. I am not educated. I also understand things late. Because of that I don't go to groups’ (non-attender, learning and developmental disability). A hearing impaired woman said: ‘I can't understand clearly what other people say. If they are speaking quite far away, I can't hear them. That's why I don't go to the group regularly’ (non-attender, sensory disability).

### Benefits of group attendance

In MIRA groups, women enjoyed learning about maternal and newborn health, and participating in group activities (stretcher schemes, picture card games, video shows, quiz contests, etc.). Many deposited money in the group maternal and child health funds. Women felt supported by group members, and attending the group gave them time to relax and socialize with friends: ‘I also went there to have fun with friends. There is a gathering of friends over there, all the women come there and to meet each other. It's fun’ (non-attender, psychological behavioural disability).

Disabled women told us that group attendance had positively affected how the community perceive them, as it demonstrated their capacity and sense of social responsibility: ‘They say: “she is also a known figure of this place”. I am working as a health and social worker. I am also working with MIRA, and the community members are interested in this, so I am treated nicely by them’ (attender, learning and development disability).

Often, savings groups needed more participants (the more participants, the more money), and therefore disabled persons were encouraged to attend. Most disabled women in savings groups liked saving money, taking loans, and could see the future benefits: ‘I can get a loan and be charged less interest and all the group members are very friendly. And if we want to do some income generating work then we can get a loan. The money we save will be useful in our old age’ (attender, intellectual disability). Participation in savings groups may also have positively affected people's perceptions of disabled women: ‘We are all saving money so they treat me equally … . I saved money at that group which will be useful for me in my difficult situation’ (attender, physical disability). Agriculture groups were also popular as they enabled women to buy cost-price fertilizer and raise animals.

### Disabled peoples' involvement in community activities

Some disabled women felt that disabled people were encouraged to participate in community activities. Several women knew disabled persons who were involved in disability-specific organizations, but these organizations generally organized training, not regular group meetings. A few women mentioned other disabled people in community groups, but most felt that there were only a few disabled people in their community and they were not routinely in contact with them.

Most participants felt that disabled persons were invited to participate in community development activities according to their ability. Much of this work is physical, for example road or temple construction. Supervisors mentioned that communities expected disabled persons to participate in these activities perhaps more than non-disabled persons, who were able to do every day work.

We also asked whether disabled persons had chairperson or secretary positions within groups. Most participants did not know any disabled person in a leadership position: ‘Disabled people are not given the chance to have a leading post in the group … we are discriminated against in regards to community work. People say we are weak we can't do anything’ (attender, intellectual disability). FCHVs felt that competition for these positions meant that disabled persons were unlikely to take them. FCHVs and Supervisors mentioned that government quotas existed and these might increase disabled leadership, but they were unable to give an example of this.

## DISCUSSION

We found no significant differences in the percentage of women reporting that they had ever attended a MIRA women's group, between non-disabled and disabled women. This was true for women with all types and severities of disability, except women with physical disabilities, who were less likely to have attended. Women with mild disabilities, and learning disabilities were likely to attend more group meetings on average than other disabled and non-disabled women. Distance to the group did not affect women as many groups were run locally. Barriers such as poverty, family support, lack of self-confidence and attendance in many groups prevented women from attending MIRA and other groups.

Quantitative analysis from Phase II showed that non-disabled and disabled group attenders were better educated than non-attenders, and tended to be literate. This is an important finding and indicates that efforts should be made to include the less educated, as they may be among the most needy. Our findings about ethnicity, disability and group attendance are interesting but difficult to explain. Ethnicity was not mentioned as a barrier to attendance in qualitative data, perhaps because most of the population were from disadvantaged ethnic groups. The fact that more women with learning disabilities attended groups is interesting and requires further exploration. Many women with learning disabilities told us that their disability did not affect their daily life, and this may help to explain their higher attendance in MIRA groups.

### Limitations

In qualitative research, we interviewed 40 disabled women, but were only able to conduct two focus group discussions with Supervisors and FCHVs. It would have been beneficial to conduct more discussions with community members.

We were unable to sample more remote clusters in our qualitative research, as we sampled clusters with more disabled women. Women living in remote areas may have more difficulties in attending group meetings, and therefore our results about physical access need further validation in remote areas. Makwanpur District has several active disability organizations and therefore findings in this District may not be easily generalized to other districts.

We asked about attendance in groups, but presence alone does not indicate full participation (Rifkin and Kangere, 2001). The participatory design of the MIRA women's group intervention may have led to more active engagement, but future research could monitor extent of participation using Participatory Rural Appraisal tools such as the spiderweb-configuration (Rifkin *et al.*, 1988; Laverack, 2007).

Defining and measuring disability is challenging, and we recognize that our disability screening tool requires further validation checks that were beyond the scope of this article. Practitioners and researchers seeking to replicate our study should consider the extent to which the tool adequately captures disability in terms of activity limitations and functioning problems. Despite these limitations, our study provides important evidence that disabled women attend community groups in this setting, and our interviews with severely disabled women suggest that the barriers to attendance for disabled and non-disabled women are similar.

It is important to consider disability in context. In Nepal, being a single woman, having a child out of wedlock, or having a childless marriage is stigmatized (Weiss, 1999; Nahar, 2010). The women in this study were married with children, yet most disabled women in Nepal are single and face a double burden of stigma (UNESCAP, 1995). Few married women without children participated in MIRA groups, due to social pressure and cultural beliefs (Houweling *et al*., 2015). The barriers to group attendance are likely to be amplified for disabled women without children, and efforts should be made to support their access to groups and reproductive health information.

### Overcoming barriers to attendance

The mistreatment of many women in our study demands action and sadly, is not dissimilar to that reported in other studies (Puri, 2011; Ortoleva and Lewis, 2012). Community groups and disabled persons organizations have an important role to play in promoting social change, and barriers to attendance such as lack of self-confidence and lack of family support need to be addressed. Actively encouraging disabled women to attend groups was effective, and it would be beneficial if facilitators and group members were sensitized about the importance of making the group and it's activities more inclusive. Involving disabled persons organizations in this orientation would be beneficial. Facilitators and group members need to work with families to raise awareness about the benefits of group attendance.

Quantitative analysis about the association between wealth and group attendance among disabled and non-disabled women is difficult to interpret, yet qualitative data suggest that poverty may be a barrier to group attendance. Additional data on individual access to resources and how this relates to group meeting logistics and dynamics may help explain how the interactions between disability and poverty affect group attendance. Previous research on MIRA funds has shown that they can discourage poorer women from joining the group (Morrison *et al.*, 2010a,b). If the fund is made a ‘community fund’ as opposed to a ‘group’ fund, this could widen access beyond group members and increase the risk pool for defaulting on loan payments.

## CONCLUSION

It is encouraging that non-disabled women were attending MIRA and other community groups as it is often assumed that disabled women are socially isolated and only reached, if reached at all, by disabled persons organizations. Our findings suggest that disabled women were not excluded from community groups, and we suggest linking interventions with disability advocacy groups to support and sustain inclusion. All women require access to information to make decisions about their reproductive health, to care for themselves and their children. Disabled women's inclusion in groups indicates that the intervention reached some of the most marginalized women, enabling them to share in the health improvements that women's group participation can bring.

## FUNDING

This work was supported by UBS Optimus Foundation Innovation Grant; UK Department for International Development [200474]; and Dr Joanna Morrison was supported by a Wellcome Trust Strategic Award (085417MA/Z/08/Z). Funding to pay the Open Access publication charges for this article was provided by the Wellcome Trust.
